# Role of Fecal Microbiota Transplantation in Managing Clostridium Difficile Infection and Inflammatory Bowel Disease: A Narrative Review

**DOI:** 10.7759/cureus.51004

**Published:** 2023-12-23

**Authors:** Haider Ghazanfar, Sameer Kandhi, Trishna Acherjee, Zaheer A Qureshi, Mohammed Shaban, Diaz Saez Yordanka, Dessiree Cordero, Siddarth Chinta, Abhilasha Jyala, Harish Patel

**Affiliations:** 1 Internal Medicine, BronxCare Health System, Bronx, USA; 2 Medicine, The Frank H. Netter M.D. School of Medicine at Quinnipiac University, Bridgeport, USA; 3 Internal Medicine, BronxCare Hospital Center, Icahn School of Medicine, New York, USA

**Keywords:** severe cdi, gut dysbiosis, fecal microbiota transplantation, clostridium difficile, inflammatory bowel disease

## Abstract

Fecal microbiota transplantation (FMT) has been emerging as an alternate treatment modality in the management of patients with dysbiosis by restoring abnormal gut microbiota composition through the transplantation of normal fecal microbiota from healthy donors. This technique has lately gained a lot of attention in the treatment of recurrent or refractory *Clostridium difficile* infection (CDI) owing to its high success rates combined with its favorable safety profile. FMT has also been attracting the interest of clinicians as a new treatment option for inflammatory bowel diseases (IBD). Here, we reviewed most of the recent advancements in the use of FMT for CDI as well as its use in the treatment of IBD.

## Introduction and background

Human gut’s microbial flora

The microbial flora of the human gut is a dynamic and diverse community of commensal bacteria, viruses, and fungi [1]. Among them, bacteria constitute a major part of over 1000 different species. More than 90% of the bacterial species belong to four major phyla: Bacteroidetes, Firmicutes, Actinobacteria, and Proteobacteria [1]. The composition is influenced by various factors, such as dietary practices, medication use, the host immune system, and the microbiome itself. Natural variations within the intestinal microbiota can result in dysbiosis when stressful conditions rapidly decrease microbial diversity. A multitude of diseases, including inflammatory bowel disorders and metabolic disorders such as obesity and diabetes type II, are significantly associated with intestinal dysbiosis [1]. This disruption in the gut microbiota balance ultimately results in the alteration of gut microflora-associated functions, including the alteration of fermentation products of carbohydrates, vitamins, and short-chain fatty acids (SCFA), along with biochemical process alterations such as loss of immune equilibrium [2,3].

Studies have shown a significant difference between the gut microbiome of healthy individuals and that of IBD patients in terms of load, density, and diversity of microflora. The patterns of dysbiosis associated with IBD patients include a decrease in commensal bacteria diversity, particularly in *Firmicutes* and *Bacteroides spp.*, and a relative increase in bacterial species belonging to *Enterobacteriaceae* [1,4]. Suppression of normal gut microbiota due to antibiotic consumption accelerates colonization with a pathogenic strain of different microbes, including *Clostridium difficile* spores. Elderly patients are more vulnerable to this effect and are more likely to develop severe infections as compared to younger patients [5].

*Clostridium difficile* infection (CDI) and inflammatory bowel disease (IBD): disease burden

Inflammation involving the colonic wall is called colitis and can result from several mechanisms, the most common of which are infections and autoimmune dysregulations [6]. Inflammatory bowel diseases encompass two main pathologies: Crohn’s disease (CD) and ulcerative colitis (UC), both of which are characterized mainly by chronic inflammation of the gastrointestinal tract but are distinguished from each other by the location of the affected GI tract, clinical presentations, imaging, colonoscopy findings, and characteristic tissue histological lesions [7]. The chronic relapsing symptoms associated with inflammation and the disrupted structure of the mucosa in IBD make it a worldwide health problem [5].

Intestinal mucosal disruption, dysbiosis of the gut microbiota, and infections are major triggers of inflammation in the gastrointestinal tract. *Clostridium difficile*, a gram-positive, anaerobic, spore-forming bacterium, is one of the major contributors to gut microbiota disruption [8]. Traditionally, CDI was commonly known as superinfection or nosocomial contamination after prolonged exposure to antibiotics in hospitalized patients, affecting the regular GI microbiota [8]. Over the past few years, there has been an increase in the incidence of community-acquired CDI cases [4]. It was estimated that in the United States, every seven per 100,000 patients become infected with *Clostridium difficile* in the hospital setting [9]. These numbers are expected to increase depending on the duration of hospital stay, intrinsic patient factors, and hygienic practices within the facility [10].

IBD was originally thought to affect mostly people of Western European descent. Still, over the past several decades, this condition has become a global health issue, mostly secondary to an increase in industrialization and modernization of lifestyles all over the world [10]. The prevalence of IBD has grown significantly over the past few decades and is now estimated to be around 250-440 cases per 100,000 people [11,12]. IBD can occur in patients of any age, but the most common age group for diagnosis is between 18 and 35 years old, with a similar distribution among men and women [10]. Patients with underlying IBD have an increased susceptibility to developing CDI. Given the similar clinical symptoms of diarrhea, abdominal pain, and low-grade fever in both IBD and *Clostridium difficile*, the diagnosis of CDI in these groups of patients can be significantly delayed [13]. Delay in the identification of the infection can lead to inappropriate use of glucocorticoids or immunosuppressive therapy. CDI should be suspected when evaluating IBD patients with apparent flares, especially those who have recently received antibiotics or those with no antibiotic use but have a history of recent hospitalization. 

The exact pathophysiology of IBD is unknown and is thought to be closely related due to external environmental factors, host genetic susceptibility, intestinal microbiota derangements, and immunological response [14,15].

## Review

This review article discusses the governing mechanism associated with fecal microbiota transplantation (FMT) along with a detailed literature review of the studies evaluating the role of FMT in recurrent CDI in IBD patients and other high-risk populations. A systemic review was performed based on Cochrane [16], MOOSE [17], and Prisma [18] guidelines. An online search of the following databases: PubMed, Scopus, Embase, Medline, Cochrane, and Web of Science was performed using the search keywords "Fecal microbiota Transplantation," Recurrent *Clostridium Difficile*," "Immunocompromised and FMT ", and "FMT and IBD". No year constraints were applied in the search. Study types included randomized controlled trials (RCTs), cohort studies and case studies (case series and case reports), nonrandomized controlled studies and nonrandomized experimental studies, meta-analysis, and systematic reviews. The subsequent 110 results obtained were screened to filter out duplicates and articles not in the English language. The authors then reviewed individual articles for relevance to the subject (FMT use in recurrent CDI and IBD management).

Fecal microbiota transplantation: an emerging treatment modality for recurrent CDI

Over the past few decades, conventional antibiotic treatment practices have significantly failed in the management and prevention of recurrences associated with CDI in more than half of the patients, thus mounting the need for alternative treatment modalities. Fecal microbiota transplantation (FMT) is an emerging alternative option in the management of patients with dysbiosis by restoring the abnormal intestinal microbiome constitution through normal fecal microbiota transplantation derived from healthy donors. FMT has also been effective in the management of patients with severe relapsing CDI [19]. Significant remission rates and a better safety profile have made it an attractive and sought-after treatment option for patients who have failed standard antibiotic therapy [20,21].

Manual FMT preparations were used for hundreds of years despite concerns about safety risks such as peripheral leukocytosis or leukopenia, proliferation in lymphoid follicles, and sometimes death [22]. However, washing fecal preparation with an automated purification system based on microfiltration with subsequent repeated centrifugations significantly reduced some of the adverse effects associated with the traditional manual preparations. FMT can be administered to the patient to the upper half of the gut via esophagogastroduodenoscopy (EGD) or nasogastric/nasointestinal tubes, or through ingestion of pills, and the lower half of the GI tract through colonoscopy (particularly for proximal colon delivery), or through rectal tubes/sigmoidoscopy/enemas, or a combined approach (for distal colon delivery) [23]. FMT installations for severe and fulminant CDI colitis could be administered via colonoscopy or oral capsule administration. The colonoscopic route is more preferred as it could utilize a larger volume of the fecal substrate. This route could also ensure proper colonic delivery in cases of concomitant atonic colon or ileus that hinder fecal material delivery to the colon by the oral route.

The effectiveness of FMT doesn’t depend on the route of administration, but the route of administration may vary with the clinical condition. Less invasive techniques, such as retention enema or naso-intestinal infusion, are usually considered safer for critically ill patient populations than endoscopic interventions. The upper GI route is usually precluded in patients with ileus [24]. FMT administration done through the lower GI tract would require a minimum amount of 200-500 ml of donor stools infused into the colon via the endoscopic channel. Upper GI tract applications, on the other hand, require fairly lower volumes of donor stool suspension (about 10 times lower, i.e., around 20-50ml) and can be given through a nasogastric, nasojejunal, or gastrostomy tube, positioning the patient at an upright 45-degree angle for four hours post-infusion to avoid the risk of aspiration into the lungs [25]. Studies have also shown multiple stool infusions to be superior to a single stool infusion [25].

It is important to have donor stool thoroughly diluted and homogenized to be administered [24,25]. After suspension with the diluent, large particulates are filtered out of the stool mixture using a steel strainer or a gauze or coffee filter [26]. Once the specimen is processed, it can be directly infused through the preferred route or centrifuged and placed into gelatin capsules, which can eventually be swallowed. The processed specimen can also be frozen for later use [24]. A single dose of FMT may be enough for the management of clostridium infection; however, dose frequencies can vary when being used for the treatment of IBD patients [25]. Following an FMT administration, clinicians are expected to follow up with the patient within 3-7 days to assess the complications and success of the procedure. The next follow-up should be within 4-8 weeks [27].

**Figure 1:**
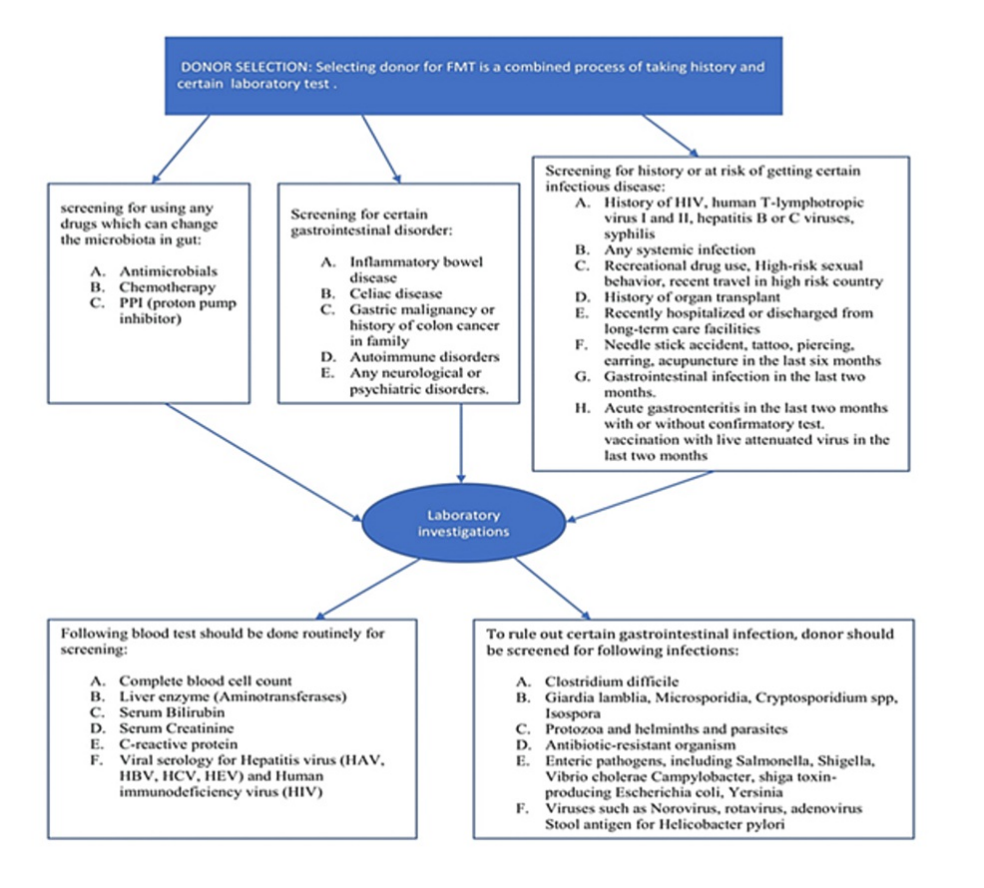
Donor selection for FMT Image Credit: Haider Ghazanfar

The most important parameters that influence the donor stool sample processing are temperature and time. Ensuring standardized and safe FMT procedures requires the recruitment of healthy donors as an essential requisite. An ideal stool donor is healthy, without any risk factors for infections or other chronic diseases and is willing to donate stool frequently if needed. The factors that should be considered for selecting donors for FMT have been presented in Figure 1 [28-30].

FMT is now an FDA-approved treatment for antibiotic-refractory *Clostridium difficile* colitis in patients with persistent colitis despite treatment with oral vancomycin, fidaxomicin, and metronidazole. FMT is also being used for recurrent and refractory cases of CDI in subjects with IBD [30].

FMT is usually a safe and well-tolerated procedure; however, common adverse effects associated with this procedure are mild and self-limiting and mainly include abdominal pain or cramps, flatulence, bloating, constipation, and transient diarrhea [31]. Adverse effects are also procedure-related, such as colonoscopic FMT, which is often known to cause nausea and vomiting secondary to the use of anesthesia for the procedure. There have also been reported incidents of minor mucosal tears and micro-perforation following biopsies obtained from regions of ischemic bowel injury in a patient with a history of chronically dilated small bowel. Upper GI FMT has long been associated with elevated risks of fecal regurgitation and vomiting [32]. Death is reported in a very minor proportion of patients undergoing procedures due to aspiration during colonoscopy [32].

Role of fecal microbiota transplantation for management of CDI in high-risk groups

FMT has gained increasing popularity as an emerging management option for IBD patients. The number of publications elaborating on the use of FMT therapy in IBD and other high-risk groups, such as immunocompromised or cancer patients, has significantly doubled over the last 10 years. A large, long-term follow-up retrospective study was carried out by Luo et al. in 2019 to assess the safety and efficacy of fecal transplantation for *Clostridium difficile* infections, particularly among high-risk subpopulations comprising immunocompromised patients with inflammatory bowel disease and those with fulminant/severe colitis with a score of 5.4 on the Charlson Comorbidity Index [33]. The overall adjusted primary cure rates of CDI observed in these patients were around 67%, with the adjusted CDI recurrence rounded off to 30% (with 90% of CDI recurrences occurring early). Common adverse events reported in the study included re-hospitalizations for recurrent infections and procedural-related events such as hypotension and intestinal perforation. The study concluded that despite the comparatively lesser primary cure rates and higher CDI recurrence rates than the lower-risk population groups, FMT therapy should still be incorporated early into the management of patients with severe or fulminant disease to avoid progression of CDI severity and recurrence [33].

Azimirad et al. performed multiple FMTs via colonoscopy in eight subjects with well-known past medical co-morbidities of IBD (seven patients with ulcerative colitis, one with Crohn's disease) and a mild to moderate disease severity index. All the subjects enrolled had a history of three prior recurrences of CDI and were refractory to conventional therapies. The cure rates observed following the first FMT were 75%, and the second FMT was 100%, respectively. One-fourth of the enrolled subjects developed chronic watery diarrhea secondary to enterotoxin-producing *Clostridium* perfringens type A strains after the second FMT. No other adverse events or IBD flare occurred in subjects [34].

Immunocompromised patients are at elevated risk of CDI. A multicenter retrospective series conducted by Kelly et al. assessing cure rates of CDI and adverse events following an FMT administration in 80 immunocompromised patients (patients treated with FMT included those with severe [34%], recurrent [55%], and refractory [11%] CDI infections) [3] with HIV/AIDS, 19 with solid organ transplant, seven with oncologic condition, 36 with immunosuppressive therapy for inflammatory bowel disease (IBD), and 15 with other medical conditions/medications) showed a CDI cure rate of about 78% after a single, with 3/4th of patients experiencing no episodes of recurrences for a minimum of 12 weeks period post-FMT. 15% of patients had to undergo repeat FMT, of which more than half had no further CDI episodes reported. The overall cure rate in the series was noted to be around 89%. Twelve patients (15%) reported serious adverse effects within 12 weeks following FMT administration, of which 10 were hospitalized. Two deaths were reported within 12 weeks of FMT, one of which resulted from a case of aspiration during anesthesia induction for colonoscopy, while the second case was not procedure related. No candidates had infections related to FMT. However, two patients were reported to have experienced unrelated infections, while five of them suffered diarrheal illness, which was self-limiting with no identifiable causal organism. Three patients reported mild abdominal pain, while five patients (14% of the IBD subpopulation) suffered a disease flare following FMT [35].

A single-center retrospective study done by Alrabba et al. evaluated the use of FMT for rCDI in 13 patients, of whom six were immunocompetent, and seven were immunocompromised (six of these immunocompromised were solid organ transplantation recipients [SOT]). The study showed that all immunocompetent patients achieved a successful cure with FMT, while three immunocompromised patients with SOT encountered failure. A second FMT in these three patients showed repeat failure in two patients and was successful in only one of them. An important predictor behind the FMT failure rates for rCDI in immunocompromised SOT recipients was noted to be antimicrobial exposure pre-FMT [36].

Cancer patients are the other well-known subset of the patient population at elevated risk of recurrent CDI infections due to multiple factors, as malignancy itself alters the immune/lymphatic/hematological systems. Cancer therapy and concomitant infections associated with frequent antibiotic use would also increase the risk of recurrent CDI (R-CDI). A retrospective study of 19 cancer patients (seven with blood-related malignancies and 12 with solid tumors; the majority with stage IV cancer; and cancer remission rates of 21%) underwent FMT for R-CDI. They found that FMT was safer and more efficacious in the management of R-CDI in about 84% of the included patients; however, the use of antibiotics in the treatment of chemotherapy/immunosuppression-related complications had significantly decreased the efficacy of FMT used in these cancer patients. Overall, no mortality or adverse events related to FMT were reported for the first month of FMT therapy [37].

In a cohort study done by Fischer et al. using data from eight academic centers, 67 IBD patients (35 with Crohn's disease and 31 with ulcerative colitis) who had undergone FMT therapy for rCDI were followed up for three consecutive months. Remission was observed in 53 (79%) patients following the initial FMT. IBD disease activity improvement was noted in 37% of patients, along with no change in 30% and worsening disease activity in 13%. Serious adverse events reported were hospitalization for IBD flare (2.9%), hospitalization for CDI (2.9%), pancreatitis (1.4%), small bowel obstructions (1.4%), colectomy (1.4%), and CMV colitis (1.4%) [38].

A meta-analysis conducted by Chen et al. evaluating the effects of FMT therapy for rCDI in patients with inflammatory bowel disease in available nine cohort studies (total patients analyzed, n = 346) till 2017 showed an initial cure rate of 81%, and the overall cure rate was reported to be up to 89%. The recurrence rate was noted to be 19%. The reported cure rates of CDI following FMT use were noted to be similar in patients, irrespective of the presence or absence of a history of IBD. (Risk ratio [RR] is almost close to one) [39].

In the systematic review done by Lai et al., 168 articles were reviewed to evaluate the characteristics of the donor, procedures, and clinical outcomes of FMT. Indications for FMT included treatment of CDI (n = 108 articles) and IBD (n = 31 articles). The overall cure rates for CDI were reported to be around 96%, and the final remission rates for UC were 39.6% and CD were 47.5%, respectively. No difference in cure rates for CDI and final remission rates for CD and UC were reported among all the available routes of administration. Adverse event rates were observed in less than 1% of cases, with the majority of them being GI-related. No difference was noted between the adverse event incidence and routes of FMT administration [40].

A summary of the above studies has been listed in Table 1.

**Table 1:** Summary of studies evaluation use of FMT in the management of rCDI in high-risk groups

Study Group and year	Study Design	Efficacy Endpoint	Adverse events	Conclusion
Luo et al. (2020) [33]	FMT for rCDI in high-risk populations: IBD, immunocompromised, Severe/fulminant colitis. (n=75)	Adjusted cure rate: 67% Adjusted CDI recurrence rates: 30%	Re-hospitalizations for rCDI.	Lower primary cure rate and higher CDI recurrence rates were seen with FMT, likely driven by higher-risk subpopulations.
Azimirad et al. (2020) [34]	Sequential FMTs use for rCDI in IBD patients (n=8) (7 UC and 1 Crohn's pt)	Cure rate after first FMT: 75% Cure rate after second FMT: 100%	Chronic watery diarrhea secondary to enterotoxin-producing (CPE) Clostridium perfringens.	Multiple FMTs are effective in significant resolution of rCDI for IBD patients. CPE-producing *C. perfringens* testing should be employed in the screening of FMT donors.
Kelly et al. (2014) [35]	FMT use for rCDI in immunocompromised patients (n=80)	CDI cure rate after single FMT: 78% Overall Adjusted cure rate: 89%	Twelve (15%) had serious adverse events within 12 weeks post FMT. Two deaths within 12 weeks of FMT. None suffered infectious complications related to FMT.	FMT use found to be effective for the management of rCDI in immunocompromised. No related infectious complications were noted in these high-risk patients.
Alrabba et al. (2014) [36]	FMT for rCDI (n=13) in both immunocompetent (6) and immunocompromised (7) pts	100% cure rate in immunocompetent group with first FMT. 3 immunocompromised solid organ recipients experienced failure with the first FMT of which 2 failed again with sequential FMT. Pre-FMT antimicrobial exposure was a predictor for failure.	No significant adverse events were noted in either group.	FMT is safe to use for rCDI with variable efficacy in immunocompromised patients. Antimicrobial exposure before FMT was an identified risk factor for FMT failure.
Ali et al. (2021) [37]	FMT for rCDI in cancer patients. (n=19)	Adjusted cure rate: 84%	No adverse events 30 days post FMT.	FMT is safer and well-tolerated in cancer patients.
Fischer et al. (2016) [38]	FMT for rCDI in IBD pts (n= 67) (35 Crohn’s and 31 UC)	Single FMT cure rate: 79%	Hospitalization for CDI (2.9%), Hospitalization for IBD flares (2.9%), SBO (1.4%), Colectomy (1.4%), Pancreatitis (1.4%), CMV colitis (1.4%).	Higher cure rates were observed when used in IBD patients.
Chen et al. (2018) [39]	Meta-analysis evaluating FMT use for rCDI in patients with IBD in 9 cohort studies (total n=346)	Overall cure rate: 89% Subgroup analysis revealed a similar efficacy in Crohn’s and UC.	IBD flares reported in 4 studies	FMT is effective for rCDI in IBD patients. No significant difference between CDI cure rates after FMT in patients with and without IBD (RR:0.92).
Lai et al. (2019) [40]	Systemic review evaluating 168 articles for clinical outcomes of FMT use for CDI (n= 108 articles) and IBD (n= 31 articles) (exclusive events)	Cure rates for CDI: 95.6% UC Remission: 39.6% Crohn’s remission: 47.5%	Overall adverse event rate: <1%	No difference in cure rates of CDI and final remission rates of IBD across all routes of FMT administration.

Role of fecal microbiota transplantation in treating IBD

A relatively large meta-analysis was conducted by Fang et al. to study the safety efficacy and protocol of FMT therapy for IBD patients, which included 459 patients across 23 cohort studies (15 studies in UC, four in CD, and four in both UC and CD) receiving FMT therapy for IBD. Primary outcomes included clinical remission rates. 28.8% of the included patients achieved clinical remission during follow-up. In comparison, the clinical response was achieved at 53%. In the pooled estimate for UC, the clinical remission was 21% vs. 30% for CD, both with a risk of heterogeneity: 10% for pediatric UC, 26% for adult UC, 45% for pediatric CD, and 22% for adult CD. Patients with moderate and severe IBD had a more significant remission from FMT as compared to patients with mild to moderate disease (p = 0.037). No impact of the delivery route on the efficacy of FMT in UC and CD was observed. In conclusion, they stated that FMT might be a potential rescue therapy and even an initial standardized therapy for IBD [41].

In a meta-analysis conducted by Narula et al. to assess fecal microbiota transplantation (FMT) as a treatment modality for active UC, four studies with 277 participants were included. FMT was associated with higher combined clinical and endoscopic short-term remission compared with placebo, with a number needed to treat of five. There was no statistically significant increase in serious adverse events with FMT compared with controls (risk ratio adverse event was 1.4) [42]. Another meta-analysis was done by Sun et al. to determine the efficacy and safety of FMT in UC, ruling out studies that included patients with infections. They assessed a total of 133 UC patients from 11 studies included in the analysis. Clinical remission (CR) was achieved in 30.4%, with no difference detected between upper gastrointestinal delivery and lower gastrointestinal delivery or between single infusions and multiple infusions (>1) of FMT. All studies reported mild adverse events [43].

Zhou et al. used the data from 16 RCTs to conduct a meta-analysis. This metanalysis aimed to study biological agents, Tofacitinib, and FMT in UC. They found that all treatments were more effective than the placebo. They did not find a statistically significant outcome in the efficacy of biological agents such as Tofacitinib and FMT. The least adverse events occurred with Tofacitinib and FMT, which are believed to be promising alternatives to biological agents with high efficacy [44].

In a randomized controlled trial, Moayyedi et al. performed a parallel study of 70 patients with active UC without infectious diarrhea, randomly assigned to FMT or placebo (once weekly for six weeks). Nine patients who received FMT (24%) and two who received placebo (5%) were in remission at seven weeks (a statistically significant difference in risk of 17%; 95% confidence interval, 2%-33%). No significant difference in adverse events was seen between the two groups. Seven out of nine patients were in remission after FMT received fecal material from a single donor. Three out of four patients with UC ≤1 year entered remission, compared with six out of 34 of those with UC > one year [20].

Xiang et al. performed a study to assess the efficacy of FMT in CD as targeted therapy for seven therapeutic targets, including abdominal pain, diarrhea, hematochezia, fever, steroid dependence, enterocutaneous fistula, and active perianal fistula. One hundred and seventy-four patients completed the long-term follow-up between October 2012 and December 2017. At one month after FMT, 72.7% (101/139), 61.6% (90/146), 76% (19/25), and 70.6% (12/17) of patients achieved improvement in abdominal pain, diarrhea, hematochezia, and fever, respectively. In addition, 50% (10/20) of steroid-dependent patients achieved steroid-free remission after FMT [45].

Sokol et al. performed a randomized, single-blind, sham (placebo surgery) study to evaluate the role of FMT in adults with colonic or ileocolonic CD after remission of flare using an oral corticosteroid. Eight patients received colonoscopic FMT, while nine patients had sham transplantation. After six weeks of steroid tapering, another colonoscopic evaluation was performed. The recipient’s fecal microbiota was not found to be similar to that of the donor’s fecal microbiota at the end of six weeks post-FMT (Sorensen index > 0.6). At 10 and 24 weeks, the steroid-free clinical remission rates were 87.5% and 50.0% in the FMT group, respectively, as compared to. 44.4% and 33.3% in the sham transplantation group. The Endoscopic Index of CD severity decreased six weeks after FMT (p = 0.03) but not after sham transplantation (p = 0.8). The absence of donor microbiota engraftment was associated with the flare. No safety signal was identified. The higher colonization by donor microbiota was associated with the maintenance of remission [46].

Caldeira et al. also conducted a meta-analysis to investigate the evidence on the efficacy and safety of FMT for IBD. Among the 60 studies included in the meta-analysis, the overall clinical remission rate was 37%, while the overall clinical response was 54%. The prevalence of adverse events was 29%. Frozen fecal material and universal donors were related to better efficacy outcomes [47].

Cheng et al. performed another meta-analysis, including 12 trials, to evaluate the efficacy and safety of FMT in CD patients. Pooled analysis showed that 62% of CD patients achieved clinical remission, and 79% of CD patients achieved clinical response post-FMT. The rate of clinical remission with fresh FMT was significantly higher than frozen FMT (73% vs. 43%). No major FMT-related adverse events were reported. Most of the reported adverse events were self-resolving [48].

Colman et al. conducted another meta-analysis to evaluate the efficacy of FMT as a treatment for patients with IBD. Eighteen studies (nine cohort studies, eight case studies, and one randomized controlled trial) were included, enrolling a total of 122 patients (79 UC; 39 CD; four IBD unclassified). Overall, 45% of patients achieved clinical remission, 22% for UC, and 60.5% for CD [49].

The frequency of FMT for the treatment of CD is still questionable. Li et al. performed a survey of 69 patients to evaluate the optimal timing for the second FMT in patients with CD. They enrolled patients with active CD who benefited from the first FMT, and then they received a second FMT to study the long-term clinical effects. In the total of 69 patients, the median time of clinical response for the first FMT was 125 days. The clinical response time to the second FMT in 56 of 69 patients was 176.5 days. They found the fecal microbiota composition closer to that of the donor ones after the first FMT. The increased urinary indoxyl sulfate, 4-hydroxyphenylacetate, creatinine, dimethylamine, glycyl proline, hippurate, and trimethylamine oxide (TMAO) after the second FMT compared to that of the baseline reflected a significant change in metabolic profiles. This study concluded that patients with CD could be administered the second course of FMT less than four months after the first FMT to maintain the first FMT's clinical benefits. This conclusion was supported by the host-microbial metabolism changes in patients with active CD [50].

CD-related inflammatory mass is one of the most challenging medical complications in the context of CD. To evaluate the efficacy and safety of multiple fresh FMTs for the treatment of intraabdominal inflammatory mass, He et al. enrolled 25 patients with CD-related inflammatory mass. Those patients received the initial FMT, followed by repeated FMTs every three months. 68.0% (17/25) and 52.0% (13/25) of the included patients achieved clinical response and clinical remission at three months after the first FMT, respectively. The proportion of patients at six months, 12 months, and 18 months achieving sustained clinical remission with sequential FMTs was 48.0%, 32.0%, and 22.7%, respectively. 9.5% of patients achieved radiological evidence of healing, while 71.4% achieved some radiological improvement. They did not report any severe adverse events related to FMT, suggesting that sequential fresh FMTs might be a promising, safe, and effective therapy to manage CD with intra-abdominal inflammatory mass [51]. 

A summary of the above studies has been listed in Table 2. 

**Table 2:** Studies evaluating the role of FMT in treating IBD

Study Group and year	Study Design	Efficacy End point	Adverse events	Conclusion
Fang et al. (2018) [41]	FMT as a therapy for IBD in 23 cohort studies (total patients, n=459) (15 studies in UC, 4 in CD, and 4 in both UC and CD)	Clinical remission rates: 28.8% Clinical response rate: 53% (21% for UC, 30% for Crohn’s)	No significant adverse events	Moderate to severe IBD patients achieve more significant remission from FMT than mild-moderate patients (P=0.037).
Narula et al. (2017) [42]	Meta-analysis assessing FMT as a treatment for active UC (n= 277)	Number needed to treat: 5	No significant increase in serious adverse events	FMT was associated with higher combined clinical and endoscopic short-term remission compared with placebo.
Sun et al. (2016) [43]	Meta-Analysis Studying FMT in Ulcerative Colitis (n= 133)	Clinical remission of UC post-FMT: 30.4%	No significant adverse events	FMT is potentially useful in UC disease management.
Zhou et al. (2021) [44]	Meta-analysis determining efficacy and safety of biological agents, Tofacitinib, and FMT in treatment of ulcerative colitis (n= 16 RCTs)	All treatments were more effective than placebo. No statistical difference in the efficacy of biological agents, tofacitinib, and FMT.	The lowest proportion of adverse events occurred with Tofacitinib and FMT	FMT has shown to be a promising alternative to biological agents with high efficacy.
Moayyedi et al. (2015) [20]	FMT as a therapy for active Ulcerative Colitis (n = 70)	Clinical remission: FMT 24% vs. placebo 5% at seven weeks	No adverse events in both groups	FMT was superior to placebo in inducing clinical remission in active UC patients at seven weeks.
Xiang et al. (2020) [45]	Efficacy of FMT in Crohn’s disease for seven therapeutic targets (abdominal pain, diarrhea, hematochezia, fever, steroid dependence, enterocutaneous fistula, and active perianal fistula)	One-month post-FMT remission rates of therapeutic targets were: Abdominal pain: 72.7%, Diarrhea 61.6%, Hematochezia 76%, Fever 70.6%, Steroid Dependence 50%		FMT can be used as targeted therapy for Crohn’s, especially for abdominal pain, hematochezia, fever, and diarrhea.
Sokol et al. (2020) [46]	Efficacy FMT in pts with colonic or ileocolonic CD. (n=17; 8 FMT vs 9 sham transplantation)	None of the patients reached the primary endpoint which is successful colonization of the donor microbiota at 6 weeks	No adverse events noted	Single FMT might not be enough to induce significant changes in patients with colonic or ileocolonic CD.
Caldeira et al. (2020) [47]	Meta-analysis - FMT for IBD. (n= 67 studies)	Clinical remission rates post FMT in IBD patients 37% (RR: 1.7) Clinical response rates post FMT: 54% (RR:1.68)	Prevalence of adverse events similar in fresh and frozen FMT: 30.2%	Frozen FMT related to a higher rate of clinical remission.
Cheng et al. (2021) [48]	Meta-analysis evaluating FMT in Crohn’s disease (n= 12 trials)	Clinical remission rates: 62% Clinical response rates: 79%	No major adverse events	FMT is an effective and safe therapy for Crohn’s disease.
Colman RJ et al. (2014) [49]	Meta-analysis evaluating FMT as a treatment for IBD (n= 18 studies)	Clinical remission rates: 45%	No major adverse events	FMT is a safe, but variably efficacious treatment for IBD.
Li et al. (2019) [50]	Evaluate optimal timing for the second FMT in patients with CD (n=69 pts)	Clinical response time to the second FMT was 176.5 days		Patients with Crohn’s could be administered the second course of FMT in <4 months after the first FMT to maintain clinical benefits.
He et al. (2017) [51]	Use of multiple fresh FMTs for Crohn’s related intraabdominal inflammatory mass (n=25)	Clinical remission at 6, 12, and 18 months with sequential FMTs was 48.0%, 32.0%, and 22.7%, respectively.	No serious adverse events	Sequential fresh FMTs are safe and effective in managing CD with intra-abdominal inflammatory mass.

Adverse events due to FMT

In 109 publications on FMR enrolling 1555 individuals, adverse events appear to be uncommon, often mild, and self-limiting [52]. In rare cases of bacteremia, perforations and death can occur [53]. Defilipp et al. described two patients with extended-spectrum beta-lactamase (ESBL)-producing Escherichia coli bacteremia after FMT. Both patients received FMT from the same stool donor employing genomic sequencing, and one of them died. Enhanced donor screening is required to decrease the chance of transmission of virulent microorganisms and lessen adverse infectious events [53].

Wang et al. performed FMT on 139 patients with mild to severe CD to evaluate the risk factors for adverse events in the long term. During one month after FMT, 13.6% of mild adverse events occurred, including diarrhea, fever, abdominal aches, flatulence, hematochezia, vomiting, bloating, and herpes zoster. No adverse events beyond one month were observed. Among the possible risk factors, they found that only fecal microbiota purification methods were closely associated with adverse events. The rate of an adverse event in patients undergoing manual procedures to prepare fecal microbiota was 21.7%, significantly higher than the 8.7% in those undergoing an automated procedure to prepare fecal microbiota. The manual or automated purification of fecal microbiota did not correlate with the efficacy of FMT [54].

In a recent study by Gianluca Ianiro et al., 17 out of the 18 patients with IBD and recurrent *Clostridium difficile* treated with FMT had a negative CDI and had significant improvement in IBD disease activity at eight weeks follow-up. No serious adverse events were observed in the study [55].

A study that included 56 CDI patients (22 with ulcerative colitis and 13 with *Clostridium difficile*) who received FMT procedures via colonoscopy was successful in 48/56 (85.7%) of cases. In contrast, over 50% of patients with UC had a sudden outbreak of IBD disease activity. The largest study on IBD patients with recurrent or refractory CDI included 67 patients; among them, 35 had *Clostridium difficile*, 31 had ulcerative colitis, and one had indeterminate colitis. Respectively, the positive outcomes in first, second, and third fecal microbiota transplantation were 79%, 88%, and 90%. After transplantation, 25 patients (37%) had improvements in IBD disease activity, 20 patients (30%) reported no change, and for nine patients (13%), the disease aggravated [56].

Some patients with IBD respond imposingly to FMT therapy, whereas other patients fail to respond; the reason is still unclear. The success of FMT in the treatment of IBD can be determined by many factors, such as host genotype, donor, the course of the disease, the use of different antibiotics linked to illness onset, and the specific types of IBD-linked dysbacteriosis. So far, FMT has proven safe and efficacious in clearing CDI from patients with IBD, but the outcomes need to be evaluated further [41-56].

## Conclusions

As discussed, FMT has been a well-used treatment modality and is recommended in the 2021 American College of Gastroenterology (ACG) and Infectious Disease Society of America (IDSA) guidelines for the treatment of recurrent CDI. FMT use in the treatment of IBD has shown great promise, especially in patients with increased risk factors for developing IBD-associated CDI. Studies have shown a decrease in rates of recurrent CDI and improved IBD disease activity in these patients. Though the exact mechanism of this is unclear, there is a potential role of gut microbiome involvement in the pathogenesis of IBD, and thus, restoration of the microbiome with FMT is shown to benefit patients with IBD. Current guidelines still recommend pharmacological management of IBD, as the safety and efficacy of FMT in IBD are not extensively studied, and patient acceptance is not high. However, more clinical trials with longer follow-ups on safety profiles are needed to assess the efficacy of FMT in the management of IBD.
